# Update to the RANO Working Group and EANO recommendations for the clinical use of PET imaging in gliomas

**DOI:** 10.1093/neuonc/noaf170

**Published:** 2025-07-28

**Authors:** Norbert Galldiks, Joerg C Tonn, Matthias Preusser, Nathalie L Albert

**Affiliations:** Department of Neurology, Faculty of Medicine and University Hospital Cologne, Cologne, Germany, Institute of Neuroscience and Medicine (INM-3), Research Center Juelich, Juelich, Germany, and Center of Integrated Oncology Aachen Bonn Cologne Duesseldorf (CIO ABCD), Germany; Department of Neurosurgery, University Hospital Munich, Ludwig-Maximilians-University, and German Cancer Consortium (DKTK), partner site Munich, Munich, Germany; Division of Oncology, Department of Medicine I, Medical University of Vienna, Vienna, Austria; Department of Nuclear Medicine, University Hospital Munich, Ludwig-Maximilians-University, and German Cancer Consortium (DKTK), partner site Munich, Munich, Germany

For decades, several amino acid PET tracers have been evaluated for improving diagnostics in patients with brain tumors, especially gliomas. In clinical routine, the most important clinical indications for amino acid PET imaging are the differentiation of neoplasm from non-neoplastic diagnoses, the delineation of glioma extent for identifying tumor areas with increased uptake intensity for diagnostic biopsy or planning of local therapies (ie, target volume definition for resection or radiotherapy), the differentiation of treatment-related changes such as pseudoprogression or radiation necrosis following radiotherapy combined with alkylating chemotherapy from glioma relapse at follow-up, and the assessment of response to anticancer agents including the estimation of survival time.^1,2^ In addition to gliomas, an increasing body of literature suggests that amino acid PET is also of considerable diagnostic value for patients with brain metastases, especially for distinguishing treatment-related changes after radiosurgery or immunotherapy using checkpoint inhibitors from recurrent brain metastases.^3,4^

More recently, on behalf of the PET/RANO group and the EANO, several newly written and updated guidelines for amino acid PET imaging in patients with gliomas and brain metastases were published. In particular, a multidisciplinary panel of experts recommended criteria for the assessment of response to neurooncological treatment based on amino acid PET parameter changes obtained from serial scans for both patients with diffuse gliomas (PET RANO 1.0 criteria) and brain metastases (PET RANO BM 1.0 criteria) enrolled in clinical trials.^5,6^ Furthermore, the almost a decade old inaugural guideline of the PET/RANO group for the clinical use of amino acid PET imaging in patients with gliomas^2^ has now been updated,^7^ also encouraged by the substantial increase in PET imaging research in gliomas, with several hundred further publications since its initial release in 2016. Furthermore, accumulating amino acid PET data indicate a considerably interest of the community in using this diagnostic imaging tool for clinical-decision making.

Nevertheless, scientific shortcomings should be considered. For instance, most amino acid PET studies have retrospective nature, often involving a relatively small number of patients, and are commonly conducted at single centers. Additionally, several PET studies rely on outdated WHO classifications of gliomas, and, despite growing evidence in the last ten years, class 1 evidence remains scarce.^7^ To achieve high-level evidence, prospective multicenter studies evaluating the diagnostic accuracy of amino acid PET with predefined endpoints are strongly needed. Furthermore, the recent development of the PET RANO 1.0 and PET RANO BM 1.0 criteria for patients with glioma as well as brain metastases, respectively, may help fostering the inclusion of amino acid PET into clinical trials and thereby the generation of prospective, systematically acquired datasets.

While amino acid PET is already widely used in large neurooncological centers in Europe according to a survey assessed by the EORTC Brain Tumor Group^8^, its use in many countries, including the USA is still restricted to only few academic centers. The recent publication of the latter mentioned guidelines for the clinical use of PET imaging in gliomas as well as the PET RANO 1.0 criteria for gliomas and PET RANO BM 1.0 criteria for brain metastases, combined with the expected broader availability of amino acid PET in near future, may facilitate prospective multicenter clinical trials—potentially incorporating PET parameter changes related to neurooncological treatment according to the PET RANO criteria as new endpoints.^9^ To this end, this may also help to overcome the lack of class 1 evidence demonstrating that incorporating PET imaging into clinical workflows may improve patient outcomes.

Providing class 1 evidence may also support reimbursement efforts, particularly in regions where statutory health insurances do not cover the costs. This is relevant in many parts of the academic landscape worldwide, including low- and middle-income countries. In Europe, reimbursement varies among countries, although cost-effectiveness analyses showing clinical and economic benefits potentially support broader coverage^10^.

As members of the PET/RANO group, we welcome efforts to increase the availability of amino acid PET imaging for patients with brain tumors and encourage its use in clinical trials and, ultimately, patient care.
